# Analysis of patient safety event report categories at one large academic hospital

**DOI:** 10.3389/frhs.2024.1337840

**Published:** 2024-04-02

**Authors:** Cody Mitchell, Logan Butler, Alexa D. Holloway, Jin H. Ra, Karthik Adapa, Caprice Greenberg, Lawrence B. Marks, Thomas Ivester, Lukasz Mazur

**Affiliations:** ^1^ Division of Healthcare Engineering, School of Medicine, University of North Carolina at Chapel Hill, Chapel Hill, NC, United States; ^2^ Division of Acute Care Surgery, Department of Surgery, School of Medicine, University of North Carolina at Chapel Hill, Chapel Hill, NC, United States; ^3^ Department of Surgery, School of Medicine, University of North Carolina at Chapel Hill, Chapel Hill, NC, United States; ^4^ Department of Radiation Oncology, School of Medicine, University of North Carolina at Chapel Hill, Chapel Hill, NC, United States; ^5^ UNC Health, University of North Carolina at Chapel Hill, Chapel Hill, NC, United States; ^6^ School of Information and Library Science, University of North Carolina at Chapel Hill, Chapel Hill, NC, United States

**Keywords:** patient safety, safety event, event reporting, TeamSTEPPPS, operating room, surgical error, communication, TENTS

## Abstract

Given the persistent safety incidents in operating rooms (ORs) nationwide (approx. 4,000 preventable harmful surgical errors per year), there is a need to better analyze and understand reported patient safety events. This study describes the results of applying the Team Strategies and Tools to Enhance Performance and Patient Safety (TeamSTEPPS) supported by the Teamwork Evaluation of Non-Technical Skills (TENTS) instrument to analyze patient safety event reports at one large academic medical center. Results suggest that suboptimal behaviors stemming from poor communication, lack of situation monitoring, and inappropriate task prioritization and execution were implicated in most reported events. Our proposed methodology offers an effective way of programmatically sorting and prioritizing patient safety improvement efforts.

## 1 Introduction

Adverse events in operating rooms (ORs) can cause physical, emotional, and financial harm. The OR is a highly complex and variable environment, and errors may arise via systems, individuals, equipment, or any combination of these factors (1). Hospital-wide voluntary patient safety event reporting systems have been developed and implemented to document and learn from these events and are essential tools for error and harm reduction (2). These systems are useful for ascertaining root causes and contributing factors associated with near-misses, unsafe work conditions, and actual patient harm (2).

Non-technical skills such as communication, leadership, situation monitoring, and mutual support are needed in successful clinical interactions and, when neglected or improperly used, can contribute to patient safety events (3, 4). Team Strategies and Tools to Enhance Performance and Patient Safety (TeamSTEPPS) is a robust framework for evaluating such skills. TeamSTEPPS is supported by the Teamwork Evaluation of Non-Technical Skills (TENTS) instrument for analyzing corresponding safety behaviors (5).

This study describes the results of an innovative study applying the TeamSTEPPS framework and TENTS instrument analysis to patient safety event reports at one large academic medical center to identify potential contributing behavioral factors.

## 2 Methods

Our academic medical center has an internal event reporting system available to all employees. It can be accessed via a web-based portal on all hospital computers or a link within our electronic health record. Employees are encouraged to report all patient safety events (e.g., harm events, near misses, unsafe work conditions). 873 reports submitted from 6 to 1-22 to 11-30-22 were reviewed. Among these, 296 reports related to the OR or perioperative areas were de-identified for further review. Discrete data extracted from each report included (i) date of the event, (ii) location within the hospital, (iii) operating service, and (iv) pre-established event type (i.e., process issues, anesthesia/pharmacy complication, count discrepancy, etc.).

Two independent reviewers received rigorous TeamSTEPPS training and a guided instruction for using the TENTS instrument (Table 1) with clinical examples. Both reviewers read each report, established frequencies for each pre-established event category in the risk management database and identified suboptimal behaviors associated with the analyzed event. Once completed, reviewers discussed and reconciled any discrepancies. If conflicts persisted, a third reviewer evaluated the data and made a final decision for the TENTS instrument analysis. The collected data was grouped and sorted based on the frequency, event category, and TENTS behaviors, with associated descriptive statistics. A data visualization chart was constructed to better understand the relationship between report category frequency and the number of contributing unique TENTS behaviors. Axes were divided in half so that each point could be grouped and studied for patterns.

**Table 1:** TENTS Instrument behaviors with numbering, grouped by broad behavior category based on the TeamSTEPPS framework.

Category	Number	TENTS Instrument Behavior
Communication	1A	Communicates and receives information appropriately
	1B	Comfortable speaking up and asking questions
	1C	Responses to feedback between team members
	1D	Communicates and receives information to/from patient
	1E	Uses language in urgent situations appropriately
	1F	Utilizes teamwork tools (e.g. huddles, closed-loop communication, periodic planning, and updates)
	1G	Learns together, focuses on improvement following a problem
Leadership	2A	Leaders effectively manage team during their roles
	2B	Verbalizes plan: intentions, recommendations, timeframes
	2C	Delegates tasks appropriately
	2D	Instructs as appropriate to the situation
Situation Monitoring	3A	Pays attention to surroundings/environment
	3B	Aware of each other, contributions, strengths, and weaknesses
	3C	Verbalizes adjustments in plan as changes occur
Mutual support/assertion	4A	Willingness to ask for help or additional resources
	4B	Willingness to supports others across different roles
	4C	Accomplishes and prioritizes tasks appropriately
	4D	Employs conflict resolution

## 3 Results

**Figure 1:**
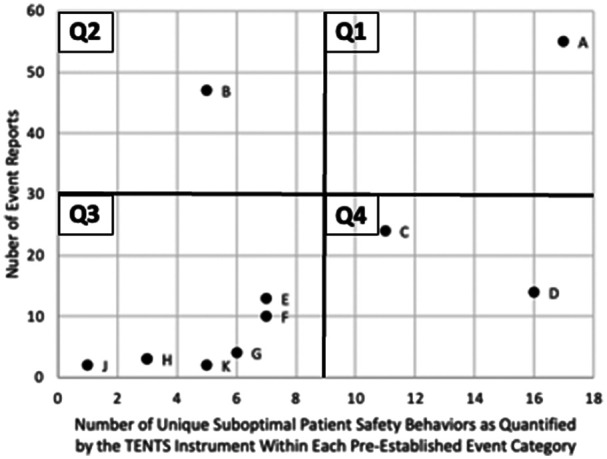
Data visualization chart. Each point represents one category of safety event reports (Table 2). Quadrants were created to compare categories based on volume of reports within the category and frequency of TENTS behaviors identified. For our analyses, axes were arbitrarily cut in half based on final charting of the data.

Of the 296 events related to the OR and perioperative areas, 177 had clearly described suboptimal behaviors identified within the report. Table 2 provides a breakdown based on adverse event category and unique TENTS behaviors. Figure 1 provides the data visualization chart summarizing the data into four quadrants, as shown.

**Table 2:** Summary of results by event report categories.

Safety Event Report Category:	Number of Reports (*N*)	Number of Unique TENTS Behaviors (*N*)	TENTS Behaviors																	
			1A	1B	1C	1D	1E	1F	1G	2A	2B	2C	2D	3A	3B	3C	4A	4B	4C	4D
A. Process/Administration Issues	55	17	25	4	4	2	3	12	5	9	9	4	0	13	4	5	2	2	37	7
B. Count Discrepancy (Instruments)	47	5	8	0	0	0	0	3	0	0	0	2	0	44	0	0	0	0	3	0
C. Improper Patient Treatment/Management	24	11	4	0	0	1	0	2	2	0	2	4	0	19	0	1	1	0	6	2
D. Interpersonal issues	14	16	10	2	5	3	2	7	1	5	2	0	0	2	2	1	2	2	9	4
E. Surgical Specimen Issues	13	7	9	0	1	0	0	3	0	0	0	2	0	6	0	0	0	1	11	0
F. Improper Consent or Surgical Site Identification Issue	10	7	3	0	0	7	0	4	1	0	2	0	0	1	0	0	0	0	3	0
G. Anesthesia Complications	4	6	2	0	0	0	1	0	0	0	1	0	0	2	1	0	0	0	2	0
H. Malfunctioning/Broken Equipment	3	3	0	0	0	0	0	1	0	0	0	0	0	2	0	0	1	0	0	0
I. Severe Patient Complications/Events	3	3	1	0	0	0	0	1	0	0	0	0	0	2	0	0	0	0	0	0
J. Intentionally Retained Foreign Objects	2	1	2	0	0	0	0	0	0	0	0	0	0	0	0	0	0	0	0	0
K. Pharmacy Complications	2	5	1	0	0	0	0	0	0	0	0	1	0	2	0	0	0	1	1	0
**Total**	**177**	-	**65**	**6**	**10**	**13**	**6**	**33**	**9**	**14**	**16**	**13**	**0**	**93**	**7**	**7**	**6**	**6**	**72**	**13**

Data is further broken down by TENTS behaviors, i.e., whether suboptimal behaviors were identified within the report. Bold letters indicate correlating point on Figure 1. Behavior number (1A, 1B…) correspond to behaviors listed in Table 1 Note that the most commonly identified behaviors within these reports were 1A, 3A, and 4C.

The three most common suboptimal behaviors contributing to adverse patient safety events among all reports were (i) “communicates and receives information appropriately” (1A: *N* = 65), (ii) “pays attention to surroundings/environment” (3A: *N* = 93), and (iii) “accomplishes and prioritizes tasks appropriately” (4C: *N* = 92), and the least common suboptimal behavior was “instructs as appropriate to the situation” (2D: *N* = 0). The most common adverse patient safety event categories were (i) Process/Administration Issues (*N* = 55) and (ii) Count Discrepancy (*N* = 47), and the least common patient safety events were (i) Intentionally Retained Foreign Objects (*N* = 2), and (ii) Pharmacy Complication (*N* = 2) (see Table 2 for breakdown by each category and TENTS behavior).

The most frequent type of event with coupled (many identified, *N* = 17) suboptimal behaviors was Process/Administration issues (Q1: top-right quadrant in Figure 1). The most frequent type of event with isolated (few identified, *N* = 5) suboptimal behaviors was Count Discrepancy (Instruments) (Q2: top-left quadrant in Figure 1). The least frequent type of event with isolated (*N* = 1) suboptimal behaviors was intentionally retained Foreign Objects (Q3: bottom-left quadrant in Figure 1). The least frequent type of event with coupled (*N* = 16) suboptimal behaviors was Interpersonal Issues (Q4: bottom-right quadrant in Figure 1).

## 4 Discussion

Results suggest that the TeamSTEPPS framework and TENTS instrument can be effectively used to analyze potential contributing behavioral factors to reported patient safety events. We constructed and visually displayed four quadrants that can aid healthcare leaders and patient safety improvement professionals in learning from our proposed analysis. The three dominant suboptimal behaviors contributing to patient safety events were (i) suboptimal communication, (ii) lack of situation awareness, and (iii) inappropriate task prioritization and execution. Below, we discuss possible learnings from each quadrant in Figure 1.

### 4.1 Q1: coupled suboptimal behavior(s) responsible for high-frequency safety events

Q1 in Figure 1 represents administrative based events that are primarily based on behaviors that are highly coupled (e.g., influence each other) and occur at a high frequency. While most of these events in our data set did not lead to patient harm, they could prove to be challenging to address as they involve many procedural tasks that require many operations and behaviors to go as planned (e.g., scheduling, cleaning and prepping ORs between cases, receiving prior authorizations for procedures). Thus, given the complexity of such operational issues, hospital leaders and improvement professionals could invest in *programmatic systems and process improvement efforts* to eliminate such inefficiencies and waste (e.g., optimization of handovers, projects to continuously eliminate waste from the system). Examples of such improvement efforts can be found in the works by Breuer (6) and Meretoja (7).

### 4.2 Q2: isolated suboptimal behavior(s) responsible for high-frequency safety events

Q2 in Figure 1 represents patient safety events primarily based on isolated behaviors that occur at a high frequency. In our data set these events are represented by errors related to the surgical count of instruments and soft goods, with the most frequent contributing behavior being the lack of situational awareness. Therefore, a targeted effort to establish and implement a *safety barrier*, which by high reliability standards is highly standardized, robust, and effective, for instruments and soft good count could be spearheaded. Examples of such improvement efforts can be noted in the works by Mullins (8), Duggan (9) and Loftus (10).

### 4.3 Q3: isolated suboptimal behavior(s) responsible for low-frequency safety events

Patient safety events within Q3 are rare and have few suboptimal behaviors implicated. The low frequency of these events might cause organizations to place little urgency on addressing such issues. However, most events in this quadrant are unfortunately associated with patient harm and, as such, are often the most severe and undergo official root-cause analysis (RCA) procedures. Interestingly, our data suggest that these events are driven mainly by the same three dominant behaviors (suboptimal communications, lack of situational awareness, and inappropriate task prioritization) and are often rooted in the lack of psychological safety *to speak up* when complications arise. Thus, efforts to improve *soft skills* (e.g., teamwork, communication, and psychological safety) could be the most relevant to deal with such events. Our recommendations are in alignment with the prior research of Mishra (11) and Gillespie (12).

### 4.4 Q4: coupled suboptimal behaviors responsible for low-frequency safety events

Patient safety events within Q4 are rare but have many suboptimal behaviors implicated. Most events in this quadrant are based on interpersonal conflicts and improper patient treatment and management and point to interpersonal issues that are more nuanced than suboptimal behaviors alone, thus often requiring the assistance of executive leadership and human resources professionals to intervene. Focus on *organizational values (e.g., trust, culture, accountability) and corrective actions* seems most relevant as means to deal with such cases. Examples of such improvement efforts can be found in works by Brenner (13) and Bleakley (14).

We recognize that all patient safety events are important and require organizational attention. When deciding which events to address first, a decision-making process is needed. Our proposed methodology offers one possible way of sorting and prioritizing patient safety efforts programmatically. One potential complication of utilizing our proposed methodology is the underreporting of patient safety events, which is a known drawback of event reporting systems (15–17). It is also crucial to employ practical categorization of reports, which would offer insight based on event type frequency and coupling of suboptimal behaviors. There is also potential for bias in interpreting patient safety behaviors while applying the TENTS instrument. Our study offset this possibility by using two independent reviewers for each report and a third reviewer to resolve conflicts.

Patient safety event reporting systems can be analyzed to determine the role of suboptimal behaviors in safety events. At one large academic medical institution, suboptimal behaviors stemming from poor communication, lack of situation monitoring, and inappropriate task prioritization and execution were implicated in most reported events. Strategic and targeted interventions can be designed to reduce the frequency of specific suboptimal behaviors and events, and it is vital to consider the effort of implementing an intervention and the impact of selected interventions on reducing patient harm and improving the culture of patient safety.

## Data Availability

The dataset will not be available as it includes sensitive safety event reporting information that cannot be anonymized with regard to the institution. Requests to access the datasets should be directed to lukasz_mazur@med.unc.edu.
